# Implementation Profiles of an Online Communication Education Program in the CHATO National Trial Nursing Homes

**DOI:** 10.1093/geroni/igaf122.528

**Published:** 2025-12-31

**Authors:** Carissa Coleman, Frances Yang, Maria Hein, Yelena Perkhounkova, Clarissa Shaw, Maria Roche-Dean, Amy Berkley, Kristine Williams

**Affiliations:** University of Kansas Medical Center, Kansas City, Kansas, United States; University of Kansas, University of Kansas Medical Center, Kansas, United States; University of Iowa, Iowa City, Iowa, United States; University of Iowa, Iowa City, Iowa, United States; University of Iowa College of Nursing, Iowa City, Iowa, United States; Kirkhof College of Nursing, Grand rapids, Michigan, United States; University of Kansas School of Nursing, Kansas City, Kansas, United States; University of Kansas, Kansas City, Kansas, United States

## Abstract

The Changing Talk (CHAT) communication education program effectively reduces elderspeak and subsequent behavioral challenges in residents with dementia in nursing homes . As part of the nationwide pragmatic clinical trial testing Changing Talk: Online Training (CHATO), a remote implementation design was developed to capture contextual factors, ensure fidelity, and identify effective implementation strategies. Nursing homes implementing CHATO were surveyed upon completion to determine implementation strategies consistent with Expert Recommendations for Implementing Change (ERIC). A national sample of 79 nursing homes from 37 states completed the survey. Remote implementation lasted three months, one month per phase (planning, intervention implementation, and a follow-up). Staff participation rates averaged 51.6% (SD = 31.3%), ranging from 3% to 100%. Latent class analysis in Mplus version 8.10 was used to determine the implementation profiles of nursing home strategies. The three-class model demonstrated the best fit (AIC=652.05, BIC=706.55, and Sample-Size Adjusted BIC=634.03) which classified implementation profiles into (1) self-paced, (2) champion-paced, and (3) leadership-paced. Fifty-four nursing homes (68%) belonged to the Self-Paced dominant class which demonstrated all staff participation, but low participation in discussions and recognition; 22 nursing homes (28%) belonged to the Champion-Paced dominant class which used champions and reminder emails, and three nursing homes (4%) belonged to the Leadership-Paced dominant class which had relatively the highest probability of all staff participation, reminder emails and texts, discussions, and public recognition. Future analysis of these profiles will be combined with MDS data to assess which implementation styles are linked to improved resident outcomes.

